# Increased peri-ductal collagen micro-organization may contribute to raised mammographic density

**DOI:** 10.1186/s13058-015-0664-2

**Published:** 2016-01-08

**Authors:** James C. McConnell, Oliver V. O’Connell, Keith Brennan, Lisa Weiping, Miles Howe, Leena Joseph, David Knight, Ronan O’Cualain, Yit Lim, Angela Leek, Rachael Waddington, Jane Rogan, Susan M. Astley, Ashu Gandhi, Cliona C. Kirwan, Michael J. Sherratt, Charles H. Streuli

**Affiliations:** Centre for Tissue Injury & Repair, Faculty of Medical and Human Sciences, University of Manchester, Manchester, UK; Wellcome Trust Centre for Cell-Matrix Research and Manchester Breast Centre, Faculty of Life Sciences, University of Manchester, Manchester, UK; University Hospital of South Manchester, Manchester, UK; Centre for Imaging Sciences, Institute of Population Health, Faculty of Medical and Human Sciences, University of Manchester, Manchester, UK; Institute of Cancer Sciences, Manchester Academic Health Sciences Centre, University Hospital of South Manchester, University of Manchester, Manchester, UK; Manchester Cancer Research Centre Tissue Biobank, University of Manchester, Manchester, UK

**Keywords:** Mammographic density, Collagen organisation, Tissue micro-stiffness, Atomic force microscopy, Breast cancer, Cancer risk

## Abstract

**Background:**

High mammographic density is a therapeutically modifiable risk factor for breast cancer. Although mammographic density is correlated with the relative abundance of collagen-rich fibroglandular tissue, the causative mechanisms, associated structural remodelling and mechanical consequences remain poorly defined. In this study we have developed a new collaborative bedside-to-bench workflow to determine the relationship between mammographic density, collagen abundance and alignment, tissue stiffness and the expression of extracellular matrix organising proteins.

**Methods:**

Mammographic density was assessed in 22 post-menopausal women (aged 54–66 y). A radiologist and a pathologist identified and excised regions of elevated non-cancerous X-ray density prior to laboratory characterization. Collagen abundance was determined by both Masson’s trichrome and Picrosirius red staining (which enhances collagen birefringence when viewed under polarised light). The structural specificity of these collagen visualisation methods was determined by comparing the relative birefringence and ultrastructure (visualised by atomic force microscopy) of unaligned collagen I fibrils in reconstituted gels with the highly aligned collagen fibrils in rat tail tendon. Localised collagen fibril organisation and stiffness was also evaluated in tissue sections by atomic force microscopy/spectroscopy and the abundance of key extracellular proteins was assessed using mass spectrometry.

**Results:**

Mammographic density was positively correlated with the abundance of aligned periductal fibrils rather than with the abundance of amorphous collagen. Compared with matched tissue resected from the breasts of low mammographic density patients, the highly birefringent tissue in mammographically dense breasts was both significantly stiffer and characterised by large (>80 μm long) fibrillar collagen bundles. Subsequent proteomic analyses not only confirmed the absence of collagen fibrosis in high mammographic density tissue, but additionally identified the up-regulation of periostin and collagen XVI (regulators of collagen fibril structure and architecture) as potential mediators of localised mechanical stiffness.

**Conclusions:**

These preliminary data suggest that remodelling, and hence stiffening, of the existing stromal collagen microarchitecture promotes high mammographic density within the breast. In turn, this aberrant mechanical environment may trigger neoplasia-associated mechanotransduction pathways within the epithelial cell population.

**Electronic supplementary material:**

The online version of this article (doi:10.1186/s13058-015-0664-2) contains supplementary material, which is available to authorized users.

## Background

Although the causative mechanisms of most breast cancers remain poorly understood, epidemiological evidence indicates that women with radiopaque tissue occupying over 60 % of the breast are three to six times more likely to develop cancer than those with predominantly radio-translucent tissue [1, 2]. As a consequence, the proportion of radio-dense breast tissue (commonly referred to as mammographic density (MD)) is an important breast cancer risk factor [3, 4]. Crucially, unlike most breast cancer risk factors, MD can be therapeutically modified. Prophylactic treatment of patients with high MD with tamoxifen or aromatase inhibitors can reduce MD and hence breast cancer risk, but unfortunately patient tolerance to such long-term endocrine therapy is low [5, 6]. As yet the structural and compositional differences between dense and non-dense breasts, and the identity of potential therapeutic targets, remain poorly defined [7].

Since fat-rich adipose tissue is radiologically translucent, MD is determined primarily by the amount of fibroglandular material (comprising cell-rich epithelial and extracellular matrix (ECM)-rich stromal tissues) [3, 8, 9]. X-ray imaging combined with haematoxylin and eosin (H&E) staining of breast tissue implicates stromal collagen fibrosis as a key factor in raised MD [10, 11]. Such perturbations in ECM homeostasis may influence epithelial cell phenotype and tumour progression [12]. Moreover, changes in collagen organisation in the tumour microenvironment, may promote tumour initiation and progression [13]. Whilst the unmodified H&E staining protocol is well-suited to characterizing breast tissue architecture, it does not specifically identify collagen [14]. Previous studies have failed to delineate the causative mechanisms and biological consequences of increased MD because of: 1) the use of methodological approaches which are unsuited to detecting specific changes in micro-scale (i.e., cellular) ECM composition, organization and tissue stiffness and 2) inadequate control of sampling with regards to age, menopausal status and/or localized variations in breast density.

**Fig. 1:**
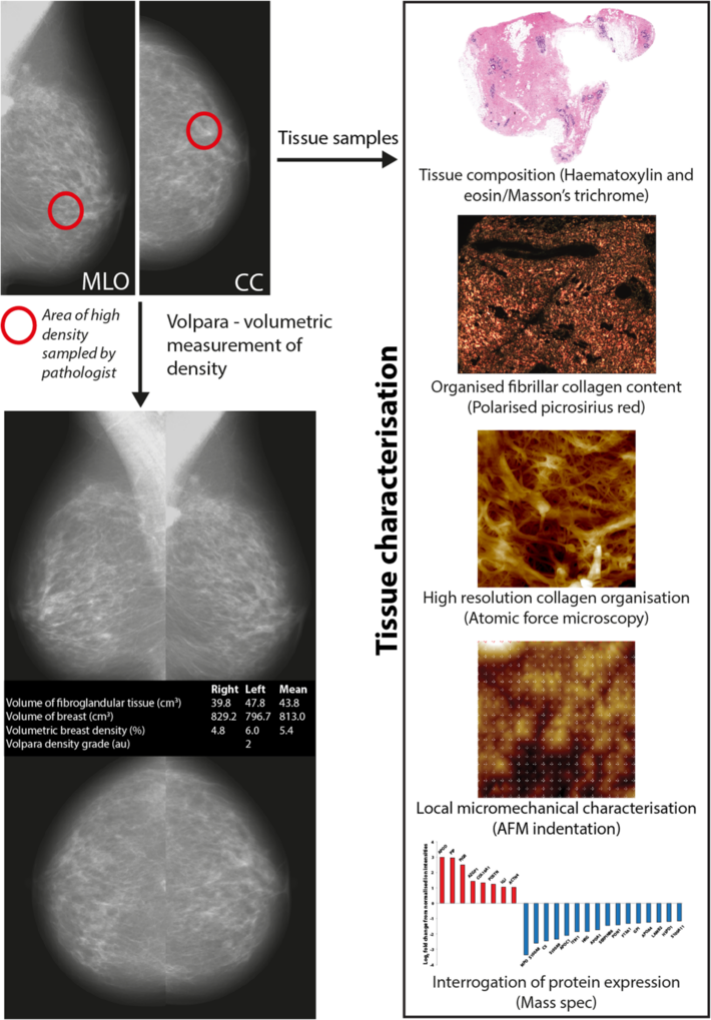
A novel workflow for sampling radio-dense breast tissue. A radiologist identified regions of elevated radiographic density in digital mammograms which were then excised by a pathologist prior to histological characterization of collagen abundance and organization in all 22 patients. *CC* craniocaudal view, *MLO* mediolateral oblique view. Following stratification of patients into low and high mammographic density (*MD*) groups (by Volpara® score), in regions of the tissue at least 4 cm from any tumours the molecular, ultrastructural and micro-mechanical drivers of MD were characterized as individuals with low (n = 6) and high (n = 6) MD by atomic force microscopy (AFM). Three of each of the samples with low and high MD (low-MS, and high-MS) were also analysed by mass spectrometry (*mass spec*). *AFM* atomic force microscopy

To overcome these shortcomings, and to define clear links between increased MD and altered breast biology, we have developed a new collaborative workflow for sampling localized regions of elevated density breast tissue from age-matched postmenopausal women. In this workflow, we have linked surgeon, radiologist, pathologist, tissue biobank, and laboratory scientist (Fig. 1). We use this workflow to test the hypothesis that increased MD in recently postmenopausal women is caused by the deposition of an ordered, and hence stiffened, fibrillar collagen matrix.

## Methods

### Cohort

Twenty-two women (54–66 years) were recruited from the Nightingale Centre breast-screening clinic. Although this Centre is one of the largest breast clinics in the UK, only about 30 suitable patients within this age range undergo surgery each year. Of these patients we were able to achieve a consent rate of 50–60 % during the duration of the study period. The local Research Ethics Committee approved the study, and all patients gave written informed consent. Women on hormone replacement therapy or who had neoadjuvant breast cancer treatment were not included in the study cohort.

### Ethics and consent statements

These studies fall under the ethics approvals for the MCRC Biobank, which is licensed by the Human Tissue Authority (licence number 30004) and has been ethically approved as a research tissue bank by the South Manchester Research Ethics Committee (Reference 07/H1003/161 + 5). Written informed consent was obtained at the time of tissue collection.

### Study workflow

All of the study participants were characterised for breast cancer risk by oestrogen receptor (ER), human epidermal growth factor receptor 2 (HER-2) and breast cancer gene (BRCA) status – none of the individuals recruited were BRCA-positive. A clinical radiologist assessed digital mammograms from each patient, and identified the area of highest radiographic density. All studies were performed on tissue situated at least 4 cm distant from any neoplasia. There is clear evidence from assessments of adipokine expression, genetic changes, mechanical properties, and radiofrequency characteristics, that tissue distant from the tumour margin will not be subject to a field change and hence can be considered to be benign [15–18]. Following surgery, highlighted tissue areas (5–10 mm^3^) were sampled from the area of high radiographic density in the resected breast, by a clinical histopathologist.

The samples were prepared for histological analysis of tissue architecture, and amorphous and aligned fibrillar collagen, by H&E, Masson’s trichrome and Picrosirius red (PSR) staining, respectively. MD was quantified by image analysis (Volpara® breast density [19]), validated against visual estimation recorded on a visual analogue scale (VAS) by a single experienced assessor. In six individuals with low and six with high MD (Table 1) micromechanical analysis was performed on sections cut from frozen tissue samples. Subsequently, ultrastructural and proteomic analyses were carried out on six tissue samples (n =3/group). Tissue samples were assessed by a histopathologist to confirm the absence of neoplasias.

**Table 1:**
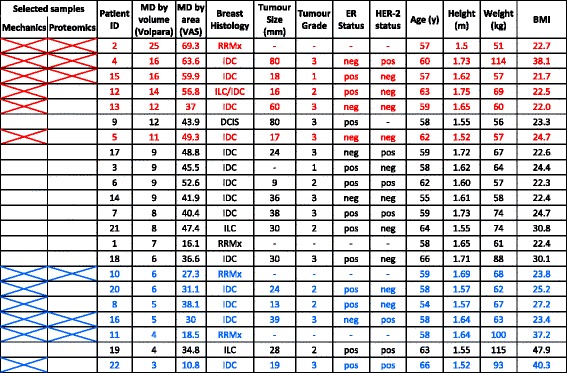
Clinical and mammographic assessment of the patient cohort

Disease status of patients assessed histologically. Also indicated are the age, height, and weight of each individual as well as the high (red) and low (blue) individuals selected for both micromechanical and proteomic analyses (crossed boxes). *BMI* body mass index, *DCIS* ductal carcinoma in situ, *HER-2* human epidermal growth factor receptor, *IDC* invasive ductal carcinoma, *ILC* invasive lobular carcinoma, neg negative, *ER* oestrogen receptor, pos positive, *RRMx* risk-reducing mastectomy, *VAS* visual analogue score

### Mammographic density

For each patient, mediolateral oblique (MLO) and craniocaudal (CC) digital mammograms were processed by Volpara® to compute the percentage of the breast volume occupied by dense tissue [19, 20]. They were also assessed by an experienced radiologist who recorded the percentage of the breast area occupied by dense tissue on a VAS.

### Tissue histology

H&E and Masson’s trichrome staining were used to assess cellularity, stromal and fat content and the abundance of amorphous collagen [21, 22]. Tissue samples were bisected, one half was fixed overnight in 4 % paraformaldehyde in PBS before processing to wax with an ASP6025 automated tissue processor (Leica, Newcastle, UK). Sections (3 μm thick) were adhered to positively charged slides, before dewaxing and methanol processing prior to staining. Following batch staining with the histology stains H&E and Masson’s trichrome, mounted sections were imaged sequentially using an SCN400 digital slide scanner (Leica, Newcastle, UK), with a Leica NA Plan Apo × 20/0.75 objective. Slides were only scanned in areas where specimen tissue was present. Resulting images (0.46 μm/pixel resolution) were saved in the MIRAX format. Image analysis was performed with custom-written rule sets for the Tissue Studio 3 software package (Definiens, Munich, Germany) [23]. More specifically, images were thresholded using the hue, saturation and brightness colour space with epithelial regions identified as the areas of lowest brightness and hue, adipose regions as areas of highest brightness and hue and stromal regions as intermediate values.

### Fibrillar collagen abundance and organisation

As ECM organisation, and not just abundance, plays a key role in determining the mechanical properties of tissues [24], we additionally stained breast tissue samples with PSR (1 h incubation with 0.1 % sirius red F3BA in saturated aqueous picric acid at pH 2 followed by clearing in 0.1 % HAc, dehydration and mounting in DPX) [25]. When visualised under cross-polarised light, the resultant collagen-associated birefringence can be semiquantitatively assessed against total tissue area [26, 27]. To clarify whether this PSR enhanced birefringence was induced by collagen fibrils alone [28], or only by assembled collagen fibrils aligned into fibres [25], we assessed the ability of PSR/polarised light microscopy to visualise aligned collagen I fibrils in young Wistar rat tail tendon, and in non-aligned fibrils reconstituted in vitro from bovine collagen I (Thermo Fisher Scientific Ltd., Loughborough, UK). Collagen fibril gels were formed at 37 °C in a hydrated atmosphere, with a gelation time of approximately 30 minutes [29]. Additional quantitative analyses of collagen orientation (coherency) were conducted on PSR stained images using a well-established methodology [105].

### Ultrastructure

We have previously shown that AFM can readily image nano-scale structural detail in unstained tissue cryosections [30]. We applied this technique to characterise the effects of MD on fibrillar collagen organisation in peri-ductal tissues, and to compare the ultrastructural architecture of collagen fibrils in rat tail tendon and reconstituted gels. The other halves of the bisected breast tissue samples, along with the rat tail tendon and reconstituted collagen fibrils were embedded in optimal cutting temperature cryo-sectioning media and snap frozen into a 2.5-cm mould with liquid nitrogen cooled isopentane [30]. Frozen sections were then cut to a nominal thickness of 5 μm in a cryostat microtome at −20 °C, air dried, washed in distilled H2O and stored at 4 °C. Subsequently these cryosections were imaged by a Bioscope Catalyst utilising Bruker’s ScanAsyst PeakForce mode (Bruker, Coventry, UK), a Bruker Scanasyst Air triangular silicon nitride cantilever (nominal spring constant of 0.2 N/m and a pyramidal tip of nominal radius of 2 nm). Height and amplitude images were captured at two scan sizes. For smaller scan areas, 10 × 10 μm was assessed at a scan frequency of 0.25 Hz and a sampling frequency of 1024 × 1024 (for a lateral spacing of 9.76 nm), whereas for large areas 150 x 150 μm was assessed at a scan frequency of 0.01 Hz and a sampling frequency 4992 x 4992 (for a lateral spacing of  30.05 nm).

### Control collagen samples

Rat tail tendon was extracted from intact tail following sacrifice of 12-month-old Wistar rats. Whole collagen fibre bundles were removed from the tail, fixed overnight in 4 % paraformaldehyde in PBS before processing to wax (as before). Sections (3 μm thick) were adhered to positively charged slides and dewaxed before staining or AFM analysis. Re-constituted collagen gels were made from Bovine Collagen I (Gibco, Warrington, UK) by treating collagen at 5 mg/mL with 1 N NaOH on ice to produce a total volume of 2 ul. This liquid suspension was placed onto a charged slide where it was allowed to dry down overnight and rinsed with distilled water before being assessed for collagen fibril abundance and organisation by both polarised light microscopy of PSR stained sections and AFM..

### Tissue micro-mechanics

Micro-indentation of rat tail tendon reconstituted collagen fibrils and peri-ductal breast tissue (from six patients with low MD and six with high MD) was carried out using 5 μm thick cryo-sections and a Bioscope Catalyst AFM (Bruker, Coventry, UK) mounted onto an Eclipse T1 inverted optical microscope (Nikon, Kingston, UK) fitted with a spherically tipped cantilever (nominal radius and spring constant of 1 μm and 3 Nm^−1^ respectively: Windsor Scientific Ltd., Slough, UK,) running Nanoscope Software v 8.15 (Bruker, Coventry, UK). The local reduced modulus was determined for each of 400 points in a 25 × 25 μm region, indented at a frequency of 1 Hz with lateral spacing of 1.25 μm. The extend curve was used in conjunction with a contact-point-based model to calculate the reduced modulus for each indentation [31]. For each biological sample, three 25-μm^2^ regions, and hence 1,200 force curves, were collected. Post hoc analyses of force curves were performed using Nanoscope Analysis v 1.40 (Bruker, Coventry, UK), whereby a baseline correction was applied to each curve before a force fit was applied using the Herzian (spherical) model and a maximum force fit of 70 %. Once all 400 force curves had been generated, quality control was applied, whereby any force values falling more than two standard deviations away from the mean value were discarded in order to account for failed indents. In general fewer than 10 % of force curves were excluded. (data not shown). Note that in the same breast samples, there was no significant difference between the reduced modulus of 5-uM-thick sections (440 kPa ± 12) and 20-uM-thick sections (450 kPa ± 17, n = 1,200, *p* >0.0001).

### Proteomics

Frozen tissue (20 mg) from three individuals with low and three with high overall MD was used to assess protein content by mass spectrometry conducted in the Faculty of Life Sciences Biological Mass Spectrometry Facility (Bio-MS). Tissues were disrupted using a Fisher 120 sonic dismembrator (Thermo Fisher, Cramlington, UK) and resuspended in 8 M urea 0.1 M Tris HCl pH8.5 [32]. The solubilised protein level was quantified using a Direct Detect system (Millipore, Billerica, MA, USA) and 25 ug were taken for subsequent digestion. Proteins were then digested using a variant of the filter-aided sample preparation (FASP) method [33] whereby proteins were solubilised in urea instead of SDS. In brief, proteins were reduced with dithiothreitol and alkylated with iodoacetamide in the presence of 8 M urea in a Micron 30 kDa centrifugal unit followed by pre-digestion with LysC in 6 M urea before digestion with trypsin in 1.5 M urea. The resultant peptides were desalted into 0.1 % formic acid in 5 % acetonitrile using Poros R3 reversed phase chromatographic media (Life technologies, Carlsbad, CA, USA) housed in 0.20-um polyvinylidene fluoride (PVDF) filter 96-well plates (Corning, New York, NY, USA). Liquid chromatography–mass spectrometry (LC-MS/MS) was performed using an Orbitrap Elite™ Hybrid Ion Trap-Orbitrap Mass Spectrometer coupled with a nano U3000 chromatography system (both Thermo Fisher, as before). The data produced was quantified using Progenesis LC-MS (Non-Linear Dynamics, Newcastle, UK) and identified using Mascot (Matrix Science, London, UK). Proteins identified by mass spectrometry (MS) were allocated to categories according to their Gene Ontology (GO) cellular compartment annotation, and enrichment of GO terms was assessed using enrichment analysis in Cytoscape (NRNB, Bethesda, Maryland, USA) [34].

### Statistical analyses

The non-parametric Mann–Whitney *U* test was applied to determine significance (Graphpad Instat La Jolla, CA, USA). Values are reported as mean ± SD. Analysis of AFM data was conducted using SPSS 20 (SPSS, Chicago, IL, USA). Normal distribution was confirmed by *q*-*q* plot. One-way analysis of variance (ANOVA) was used to compare means. For all analyses, a value *p* <0.05 was considered statistically significant.

## Results and discussion

### Patient cohort and assessment of MD

A cohort of 22 postmenopausal women (59.7 ± 3.2 years) was recruited (Table 1). Compared with previous studies, this cohort was well-defined with regards to age and menopausal status [10, 11]. In 18 patients, cancers were diagnosed by a combination of clinical and radiological investigation (bilateral mammography and ultrasound scanning). Histological confirmation of malignancy in these individuals was obtained from a clinically derived image-guided core biopsy. No patients had metastases or cancer cell involvement with the breast skin or chest wall. For this study, we only analysed biopsies that were confirmed to be neoplasia-free by a clinical histopathologist following examination of H&E and Masson’s trichrome stained sections. The samples studied were on tissue situated at least 4 cm distant from any neoplasia. Four women did not have a diagnosis of breast malignancy, and were undergoing bilateral risk-reducing mastectomy.

**Fig. 2:**
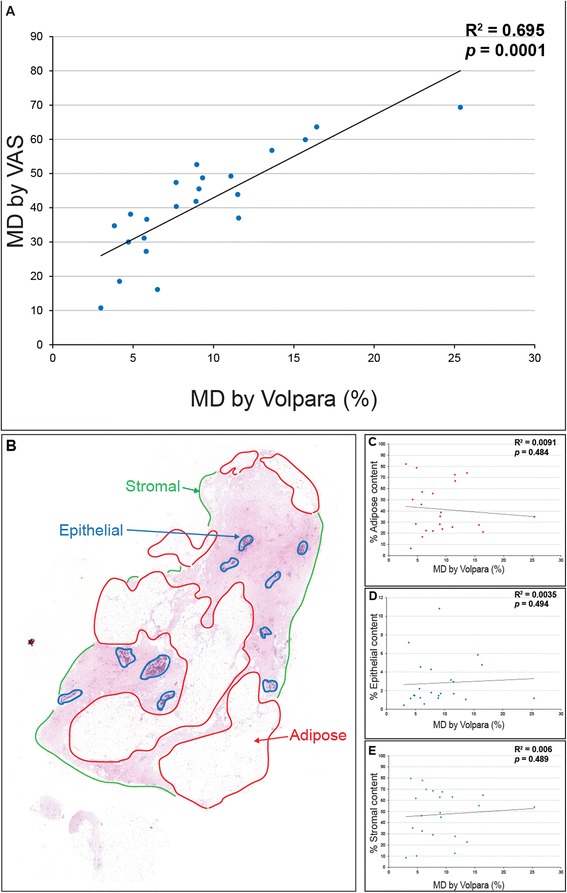
Assessment of mammographic density and tissue architecture. **a**, **b** There was a significant correlation between two common methods of assessing mammographic density (*MD*), visual analogue scale (*VAS*) and Volpara® (**a**). **b** H&E-stained paraffin section of resected breast tissue (low MD). Adipose, epithelial and stromal tissues are highlighted in *red*, *blue* and *green* respectively. **c**–**e** Tissue architecture (adipose, epithelial and stromal content) was not significantly correlated with MD

An experienced consultant breast radiologist assessed MD. Density ranged from 11–69 % using a VAS, while percent breast density measured by automated volumetric breast density software (Volpara®) varied between 3 % and 25 % (Table 1). There was a significant correlation between these two methods of measuring MD (*r*^2^ = 0.70, *p* <0.001) (Fig. 2a). Volumetric approaches for measuring breast density (as implemented by Volpara®) have been extensively validated against objective reference standard measurements obtained from magnetic resonance imaging (MRI) [35, 36] and the potential benefits of using multiple measures of MD have previously been highlighted [4]. In our study, the VAS and Volpara® approaches identified the same individuals with high MD (Table 1: patients 2, 4 and 15). Moreover, there was no significant difference between MD (as measured by either technique) in women diagnosed with a tumour and those who were tumour-free, or significant correlation between MD and tumour size (data not shown). In addition, MD was also unrelated to tumour size (MD by Volpara®, *r*^2^ = 0.150, *p* = 0.57; MD by VAS, *r*^2^ = 0.018, *p* = 0.95, data not shown).

Together these studies identified regions of normal, non-neoplastic breast tissue of varying MD, which were used for further analysis. This element of the study design is key as it demonstrates that we are assessing normal tissue rather than tissue remodelled by tumour cells. Moreover, the expression of adipokines and fatty acids is reported to differ significantly in the peri-tumour area compared to distant tissue [15], indicating that sampling regions 4 cm distant from the tumour will ameliorate any effect of the tumour on the normal tissue sampled. Also, genetic changes occurring distant from HER2-amplified breast cancers result from tumour cell contamination rather than a field change [17]. Body mass index (BMI), a strong indicator of overall adiposity and therefore breast fat content, was not significantly correlated with MD in our cohort, suggesting that adipose content is not a mediator of MD in these individuals (Table 1).

### MD correlates with collagen birefringence

To assess the potential role of stromal and parenchymal remodelling in driving increased MD, we quantified epithelial, adipose and stromal content in H&E-stained sections using automated intensity segmentation (Fig. 2b–e). Although the proportion of these tissues varied markedly between individuals (epithelial: <1 % to >10 %, adipose: <10 % to >80 %, stromal: <20 % to >80 %) there was no evidence of a relationship between tissue micro-architecture and MD (*r*^2^ = 0.0004 to 0.0053, *p* = 0.484 to 0.494). These observations contrast with the findings of previous studies, which reported a positive correlation between MD and fibroglandular (epithelial and stromal) content in the breasts of pre- and postmenopausal women aged 20–83 [9] and 40–82 years [8], respectively. By controlling for age and menopausal status, our study suggests that in the absence of larger-scale remodelling events, loss of stromal ECM homeostasis may drive changes in MD.

**Fig. 3:**
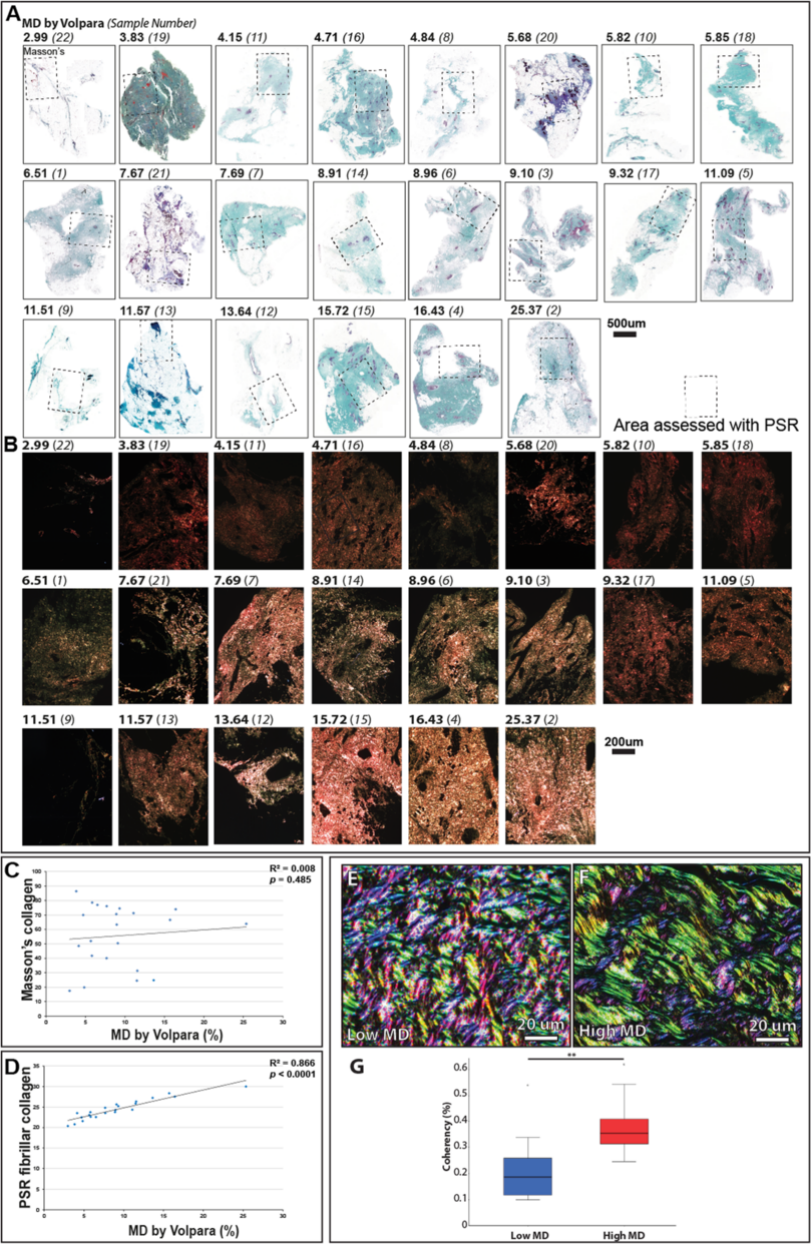
Relationship between mammographic density and amorphous and fibrillar collagen contents. **a** Masson’s trichrome staining (amorphous collagen: *blue/green*, epithelial tissue: *blue/black*). *Bold numbers* denote Volpara score®, anonymous patient identifiers are reported in *parentheses*. **b**, Serial paraffin sections stained with Picrosirius red (PSR) and visualised by polarised light microscopy. Note that the *boxed regions* (**a**) correspond to the areas corresponding to the enlarged PSR regions in **b**. For each patient, three regions of the tissue shown in **a** were analysed in more detail by PSR staining and by Masson’s trichrome staining. However for clarity, only one region is shown in **a** and **b**. **c** Amorphous collagen content as visualized by Masson’s trichrome staining was not correlated with mammographic density (*MD*). Note that for the quantitative data provided (**c** and **d**), we analysed (as above) three regions of tissue for each sample, though for clarity only one region is shown in **a**. **d** Fibrillar collagen content was significantly correlated with MD. **e**, **f** Polarised light microscopy of PSR-stained sections assessed using OrientationJ: **e** low MD, **f** high MD. Colour is indicative of fibre alignment. **g** Coherency of organised fibrillar collagen was significantly increased in high MD (0.38 % ± 0.11) compared to low MD (0.21 % ± 0.11, *p* <0.001, n =18)

Previous studies have identified stromal collagen deposition, as quantified by Masson’s trichrome [37] or H&E staining [10] as a mediator of MD. However, in the present cohort (Fig. 3a, b), we could find no correlation between MD and collagen content as determined by Masson’s trichrome (*r*^2^ = 0.0053) (Fig. 3c). This disparity may again be due to the contrasting age and menopausal status of the patients in the studies. In contrast to the narrow age range (12 years) in our cohort, others have drawn their tissue samples from individuals aged 15–90 years [37]. Moreover, in another study using individuals of a 54–75-year age range, collagen abundance was assessed using a semiquantitate scoring technique on tissue sections stained with non-specific H&E [10]. It is important that this architecture may not be maintained in regions of lower local radiographic density.

Although Masson’s trichrome is thought to specifically stain amorphous collagen, it is generally accepted that PSR-enhanced birefringence in tissue sections is specific for fibrillar collagens only [22, 25]. Here, we identified a significant positive correlation between MD and localized, PSR-enhanced, collagen birefringence in peri-ductal stroma (i.e., within 20 μm of the ducts) (Fig. 3d and D: *r*^2^ = 0.8658, *p* <0.0001), but not in perilobular or distal stroma (data not shown, perilobular: *r*^2^ = 0.1006, *p* = 0.33; distal stroma: *r*^2^ = 0.2381, *p* = 0.14). Together, these results suggest that the extent of patient MD in non-neoplastic tissue correlates with the amount of collagen birefringence revealed by PSR staining.

### Collagen birefringence correlates with fibril alignment

**Fig. 4:**
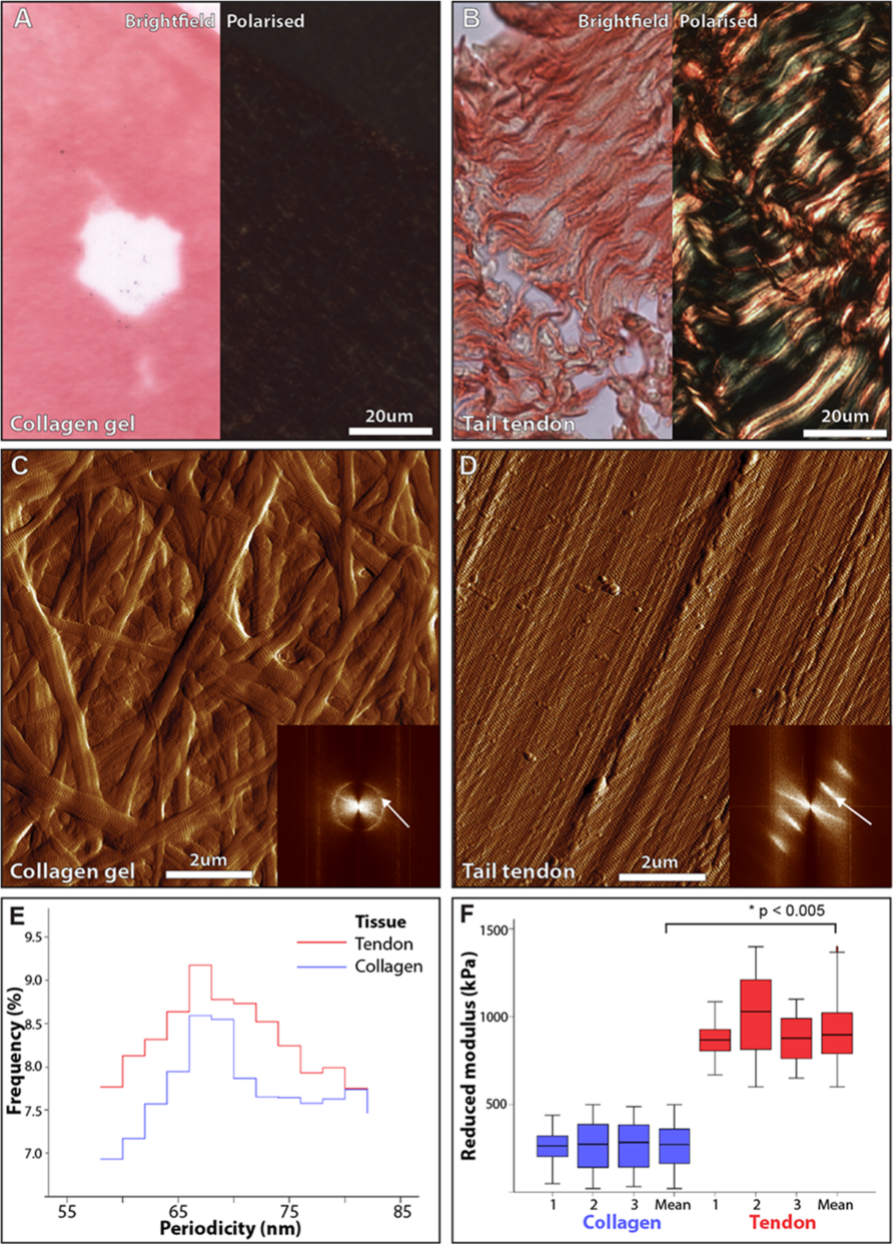
Picrosirius red-enhanced collagen birefringence in non-aligned collagen gels and aligned tendon collagen. **a**, **b** Bright field (*left*) and polarised light microscopy (*right*) of PSR-stained cryosections cut from reconstituted collagen gels (**a**) and rat tail tendon (**b**). **c**, **d** Atomic force microscopy (AFM) height maps of collagen gels (**c**) and tendon (**d**). Collagen alignment and periodicity (*white arrows*) can be readily determined from fast fourier transform (FFT) of AFM amplitude images. *Inset* position of the fast fourier transform (FFT) signals corresponding to collagen periodicity (*diffuse circles* for the gel and *discrete lines* for the tendon) are indicated by *arrows*. **e** AFM-derived collagen periodicity. **f** Micro-mechanical stiffness (modulus (MPa)) of in vitro (gel) (255 kPa ± 0.91) and in vivo (tendon) assembled collagen (869 kPa ± 0.90, *p* <0.005)

In common with many ECM structural proteins, fibrillar collagens (in particular collagens I and III) function as supra-molecular assemblies where monomers form fibrils, which in turn form fibres [13]. To characterize the effects of collagen organization on PSR-enhanced birefringence, we stained and visualized both collagen gels and rat-tail tendon cryosections. Despite being composed entirely of collagen I, PSR-stained collagen gels exhibited minimal birefringence under polarised light (Fig. 4a). In contrast, PSR-stained rat-tail tendon collagen was highly birefringent (Fig. 4b). Given the similarities in composition between the two systems, we reasoned that PSR-enhanced birefringence is critically dependent on either macro-molecular assembly of collagen monomers into fibrils, or supra-molecular assembly of fibrils into fibres. As the spatial resolution of conventional optical microscopy is insufficient to visualize individual collagen fibrils, we imaged collagen gel and tendon collagen cryosections by AFM [30]. Rather than containing amorphous collagen, both gel and tendon were composed of collagen fibrils (readily identified by their characteristic gap/overlap banding pattern) with similar periodicities of approximately 66 nm (inset in Fig. 4c and d and panel e). However, whilst the birefringent-negative fibrils in the gel were both heterogeneous in diameter and non-aligned, the birefringent-positive tendon fibrils were homogeneous in diameter and highly aligned (Fig. 4f). These results suggest that the PSR-enhanced collagen birefringence arises from fibril organisation with regards to its diameter and/or alignment, rather than its abundance.

We further explored this potential relationship by directly measuring the coherence (alignment) of birefringent collagen within peri-ductal regions in tissue sections taken from six patients with low and six with high MD (Fig. 3e, f) [105]. Collagen coherency was significantly higher in patients with high MD (Fig. 3g, Additional file 1: Figure S1). These results fit with previously published observations of tumour-associated collagen signatures in breast tumours, which suggest that ECM organisation is altered at the tumour-stroma boundary in more malignant tumours [100].

### Peri-ductal tissue contains large-diameter, stiff collagen fibres

**Fig. 5:**
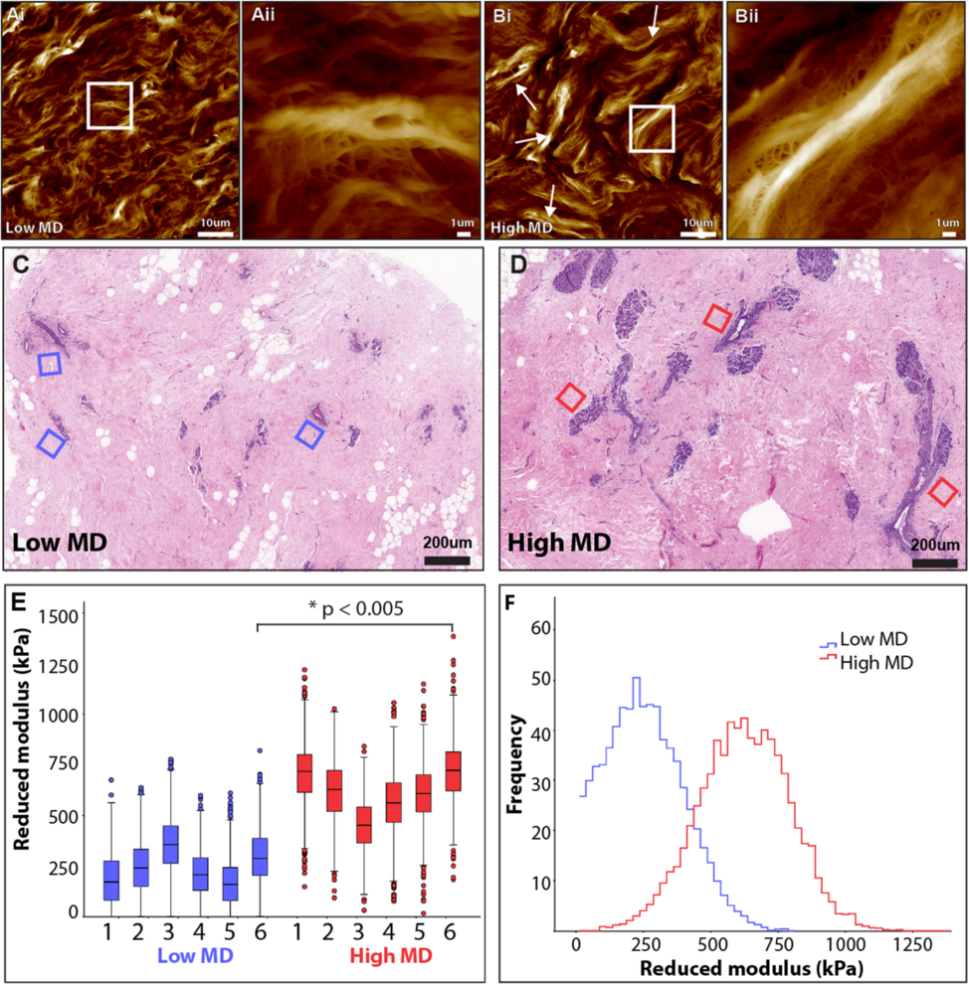
Ultrastructure and micro-mechanical stiffness of peri-ductal tissue in patients with low and high mammographic density (*MD*). **a**, **b** Atomic force microscopy (AFM) height maps of tissue of low (**a**) and high (**b**) MD captured at 150 × 150 μm and a sampling frequency of 4992 × 4992. *Arrows* indicate collagen fibril bundles (fibres) in high MD breast tissue. **aii**, **bii** Magnified regions corresponding to the *boxes* in **ai** and **bi**. Low MD tissue, as depicted (**ai** and **aii**) was characterised by the presence of loose fibrillar collagen bundles. However **(bi**, **bii**), the central large fibrillar bundle is connected to surrounding tissue by a network of fine fibrils (150–450 nm diameter). **c**, **d** H&E-stained breast biopsies from patients with low and high MD. Mechanical data were measured by atomic force microscopy (AFM) indentation (20 × 20 points) in 25-μm^2^ peri-ductal regions, shown in *blue* (low MD) and *red* (high MD) *boxes*. **e**, **f** Peri-ductal regions were significantly stiffer in patients with high MD compared with low MD (n = 12 patients, *p* <0.005)

Having identified localized PSR-enhanced birefringence as the key marker for raised MD, we used AFM imaging and force spectroscopy to determine if it correlated with collagen fibril reorganization and local micro-stiffness. Peri-ductal collagen fibrils in breast derived from patients with high, but not low, MD were organized into aligned fibrous bundles (Fig. 5a, b). The bundles were evident in tissue samples from each of the patients with high MD and were remarkably homogenous in morphology (approximately 100-μm-long and 10-μm-wide; Fig. 5bi). They were connected to surrounding material by a network of smaller-diameter fibrils/fibres (Fig. 5bii). However, few comparable bundles were present in the peri-ductal tissue of individuals with low MD (Fig. 5ai). Tissue from these individuals was characterised by the presence of loose collagen aggregates (Fig. 5aii). These findings are consistent with studies suggesting that collagen fibrils are best resolved dehydrated, with rehydration performed when nano-mechanical measurements are required [39, 40].

Fibrillar collagens are important mediators of tissue tensile strength and stiffness, while changes in tissue mechanical properties are associated with cancer initiation and invasion [41–44]. We therefore assessed the functional consequences of peri-ductal collagen remodelling in six patients with high and six with low MD using localized AFM indentation of tissue cryosections (Fig. 5c–f). Mean reduced modulus (and hence localized tissue stiffness) varied between 100 kPa and 820 kPa (median 247 kPa) in patients with low MD, compared with 200 kPa to 1380 kPa (median 611 kPa) in patients with high MD (Fig. 5e). These values are in agreement with recent work showing that when indented at lower loading rate, tissues with a high collagen fibre content have a reduced modulus in the range of hundreds of kilopascals [45]. In this analysis we utilised 5-μm-thick cryosections. Although these are relatively thin sections, it is widely accepted that if the indentation depth is smaller than 10 % of the sample thickness, the effect of the substrate can be ignored [46]. In this study the indentation depth was 20 nm or less (as is required by PeakForce QNM: [90, 91] and we observed no statistical difference in reduced modulus between 5-μm-thick and 20-μm-thick sections (data not shown). The absolute modulus values that we report are however, considerably higher than those reported for fresh breast tissue [92, 93]. These disparities may be attributable to many factors including sample preparation (freezing and dehydration), probe characteristics (we use a large diameter tip, which is unlikely to puncture soft tissue [94] and the depth and location of the indentation (our cryo-section technique allows us to measure localised mechanical changes within the peri-ductal region).

Crucially however, whilst it is well-established that absolute values for indentation moduli are strongly influenced by the specific experimental conditions (even for the same tissues [95–97], our AFM system measures relative modulus values which are: 1) comparable with previous studies on the micro-mechanics of tendon [98] and skin [99] and 2) consistently and significantly higher in peri-ductal breast tissue of high MD. The cause of this peri-ductal stiffening may be due to the influence of differential crosslink formation between the two groups as a factor in local breast tissue stiffness, however, previous studies both in vitro (collagen scaffold formation) and in vivo (tendon stiffness) have shown that collagen fibril structure and organisation mediates the mechanical properties of materials [47, 48]. Collectively, these results suggest that the increased stiffness of breasts of high MD is associated with remodelling of collagen fibrils into large-diameter peri-ductal stiff fibres, rather than the fibrotic deposition of additional collagen.

### High MD is associated with an increased abundance of collagen-organising proteins

**Fig. 6:**
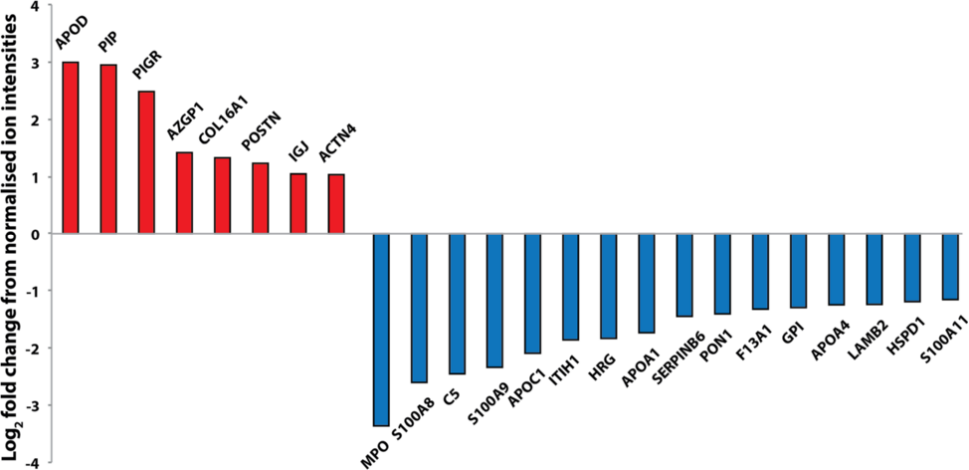
Proteomic analysis of low and high mammographic density (MD) tissue. Proteins exhibiting >2-fold difference in abundance between tissue samples derived from patients with low and high MD. *Red bars* indicate greater abundance in tissue from patients with high MD: apolipoprotein D (APOD), a breast cyst fluid component and potentially a progesterone transporter [65], which is expressed in ductal carcinoma [66]; prolactin-inducible protein (PIP), a fibronectin-degrading aspartyl proteinase [67], which is frequently expressed in androgen receptor-positive breast tumours [68]; polymeric immunoglobulin receptor (PIGR), an epithelial cell-surface-located [69] biomarker of metastatic breast cancer [70]; zinc-alpha-2-glycoprotein (AZGP1), which stimulates lipolysis in adipocytes and is expressed in up 50 % of human breast cancers [71]; collagen XVI alpha 1 chain and periostin (COL16A1 and PSTN), extracellular matrix (ECM)-regulating proteins, which control collagen fibril interactions [50–52]; and two further proteins: immunoglobulin J (IGJ) and ACTN4, which have no known links to cancer or ECM remodelling [72, 73]. Proteins with a greater abundance in low MD tissue include: myeloperoxidase (MPO), serum markers of breast cancer including the neutrophil activity marker myeloperoxidase [74]; S100A8 and S100A9 (proinflammatory regulators [75, 76]), which play an as-yet poorly defined role in metastasis [77, 78]; C5 (a proteolytic degradation product of complement C5 [79], S100A11), which facilitates keratinocyte differentiation; apolipoprotein C-I (APOC1), an inhibitor of lipoprotein/LDL receptor binding [80], which may promote chronic low-grade inflammation and breast cancer [81]; inter-alpha-trypsin inhibitor heavy chain H1 (H1ITIH1), a hyaluronan binding protein [82], which is implicated in inflammation and downregulated in breast cancer [83]; HRG, histidine-rich glycoprotein, which inhibits tumour vascularisation [84, 85]; apolipoprotein A-I (APOA1), which is reported to be protective against breast cancer [86]; SERPINB6, an ECM protease inhibitor [54]; coagulation factor XIII A chain (F13A1), which inhibits degradation of collagen precursors [53]; glucose-6-phosphate isomerase (GPI), which modulates cancer cell phenotype [87]; apolipoprotein A-IV (APOA4) - blood plasma levels are significantly reduced in BRCA1 mutation carriers modulate [88]; laminin subunit beta-2 (LAMB2), a component of basement membranes, which is implicated in tumour angiogenesis [89]. Both serum paraoxonase/arylesterase 1 (PON1) and mitochondrial 60 kDa heat shock protein (HSPD1) appear to be unrelated to either matrix homeostasis or tumourigenesis

To identify potential causative mechanisms of raised MD, we used mass spectrometry to characterise the proteomes of resected breast tissue from the patients with low and high MD (n = 3/group). Proteomic analysis of ECM has been done in a few other tissues [106]. Moreover, an extensive set of ECM proteins has been revealed within rat mammary gland preparations [101, 102], and in tumours arising from MDA-MB-231 cell lines [103]. We have identified a significant number of similar ECM proteins within the human breast (Additional file 2: Table S1), and compared the proteomes of breast tissues isolated from different human samples with either low or high MD (Fig. 6, Additional file 3: Table S2). Eight proteins with raised abundance were present in tissues from patients with high MD, and a further sixteen proteins were less abundant. Although the analysis identified proteins that are markers of breast and other cancers, there was no proteomic evidence of a relationship between MD and collagen fibrosis.

Neo-collagen fibrillogenesis is associated with expression of proteins such as the collagen I and III alpha chains, collagen processing enzymes BMP-1/tolloid C-proteinases and ADMATS N-proteinases, and regulators of fibrillogenesis including collagen V and XI, integrins α5β1 and α2β1, tenascin-X, thrombospondin 2, cartilage oligomeric matrix protein, matrilins, perlecan and the small leucine-rich proteoglycans [49]. Instead, in tissue derived from patients with high MD in our study, mass spectrometry revealed increased levels of proteins that control collagen fibril interactions, diameter and crosslinking, including collagen XVI and periostin (1.3-fold and 1.2-fold increase, respectively) [50–52]. Collagen XVI and periostin have previously been detected in breast ECM, but this is the first time that their levels have been found altered in high vs low human MD breast tissue [101, 103]. In addition there was a decreased abundance of the ECM protease inhibitors Serpin B6 and coagulation factor XIII A [53, 54].

Collagen XVI is a FACIT collagen that interacts with cells, and other ECM components including elastic-fibre-associated proteins [55]. Although this adaptor protein, which connects and organises large fibrillar networks, has not previously been associated with breast cancer, upregulation of collagen XVI is associated with cell invasiveness and proliferation in glioblastoma and oral cancer [56, 57]. Moreover, the heparin-binding glycoprotein periostin, which is commonly found in tissues under high mechanical load, regulates collagen fibril morphology and potentially fibril crosslinking via BMP1- mediated activation of LOX [50, 52, 58, 59]. Periostin is overexpressed in most breast cancers, where it enhances angiogenesis and tumour progression, and recruits Wnt ligands to maintain cancer stem cell maintenance [60, 61]. Thus, proteomic analysis reveals that high MD is associated with the *de novo* expression of proteins that are involved with ECM remodelling and which might contribute to a cancerous phenotype.

## Conclusions

Here we provide initial data showing that, in a recently postmenopausal patient cohort, neither large-scale tissue remodelling between epithelial and stromal tissues, nor collagen deposition as assessed by both Masson’s trichrome staining and mass spectrometry, correlates with MD. Instead, the peri-ductal regions of high MD breast tissue are characterized by: 1) the remodelling of collagen fibrils to form large collagen fibres, evident from both polarised light microscopy of PSR-stained sections and AFM, and 2) the upregulation of collagen-organising molecules, which have previously been identified as markers of breast cancer and metastatic colonisation rather than breast cancer risk [60, 62, 63]. Thus, the architectural remodelling of fibrillar collagens, rather than collagen fibrosis, may be a key molecular driver of raised MD, although other ECM remodelling events may also play a role. We suggest that this organisation of collagen fibrils into collagen fibres in turn increases the local mechanical stiffness of breast stroma. Although we do not yet know how collagen reorganisation into fibres in normal breast tissue may promote tumour initiation, organised collagen is a feature of human breast cancer. Indeed, collagen alignment within advanced cancer (e.g., the tumor-associated collagen signature-3 TACS-3 tissue architecture) is associated with disease outcome [104]. Given the limitations of the current study with regards to the relatively small size of the patient cohort, the inclusion of tissue from breasts that contain tumours and the highly localised nature of the mechanical mapping, further studies on the molecular causes of raised MD are clearly warranted.

Finally our data demonstrate that PSR, which is a commonly used collagen stain, is specific not for collagen fibrils, as was previously assumed, but for larger-scale organised collagen bundles. Given the impact of fibrotic diseases on human health, this ability to distinguish between structurally and functionally distinct forms of collagen has important implications for the interpretation of aberrant remodelling both in breast and in diverse fibrotic tissues [64].

## Additional files

Additional file 1: Figure S1. Coherence of collagen organization in patients with low vs high mammographic density (*MD*). **a**, **b** Polarised Picrosirius red images (×20) of peri-ductal stroma in an individual with low MD (**a**) and high MD (**b**). **c**, **d** Areas assessed by OrientationJ in individuals with low MD (**c**) and high MD (**d**). *Colour coding* is indicative of angle of collagen orientation. **d** Coherency values for six individuals with low vs six with high MD. (EPS 47751 kb) Additional file 2: Table S1. Breast extracellular matrix (ECM) proteomic data. This table shows the ECM proteins detected in our mass spectrometry analysis of human breast tissue, plus those identified by mass spectrometry in rat mammary tumours, and the ECM of tumours resulting from the in vivo growth of the MD-MBA-231 breast cancer line. *Green boxes* indicate proteins that were identified in the analyses, *red boxes* show proteins not identified. The differences in expression may arise because completely different samples have been used, e.g., human vs rat, and normal human breast tissue vs a cancer line model. (DOC 124 kb) Additional file 3: Table S2. Peptide frequencies for individual proteins identified in the mass spectrometry analysis. This table includes accession number, gene name and raw expression values for low and high mass spectrometry (MD) samples. (DOC 208 kb)

## Abbreviations

AFM: atomic force microscopy
BRCA: breast cancer gene
CC: craniocaudal
ECM: extracellular matrix
ER: oestrogen receptor
HER-2: human epidermal growth factor receptor 2
MD: mammographic density
MLO: mediolateral oblique
PBS: phosphate-buffered saline
PSR: Picrosirius red
VAS: visual analogue score
