# Supplementary material for: Increased peri-ductal collagen micro-organization may contribute to raised mammographic density
Source: Breast Cancer Res. 2016 Jan 8;18:5. doi: 10.1186/s13058-015-0664-2 (PMC4706673; doi:10.1186/s13058-015-0664-2)
Supplement: Additional file 2: Table S1. — Breast extracellular matrix (ECM) proteomic data. This table shows the ECM proteins detected in our mass spectrometry analysis of human breast tissue, plus those identified by mass spectrometry in rat mammary tumours, and the ECM of tumours resulting from the in vivo growth of the MD-MBA-231 breast cancer line. Green boxes indicate proteins that were identified in the analyses, red boxes show proteins not identified. The differences in expression may arise because completely different samples have been used, e.g., human vs rat, and normal human breast tissue vs a cancer line model. (DOC 124 kb) [file 13058_2015_664_MOESM2_ESM.doc]

Table S1

|  |  | **ECM proteins identified in** | | |
| --- | --- | --- | --- | --- |
| **Protein type** | **Protein ID** | **This study** | **Rat models [101,102]** | **231 tumours [103]** |
| *Collagens* | C1A1 |  |  |  |
|  | C1A2 |  |  |  |
|  | C3A1 |  |  |  |
|  | C5A1 |  |  |  |
|  | C5A2 |  |  |  |
|  | C5A3 |  |  |  |
|  | C5A4 |  |  |  |
|  | C6A1 |  |  |  |
|  | C6A2 |  |  |  |
|  | C6A3 |  |  |  |
|  | C6A6 |  |  |  |
|  | C7A1 |  |  |  |
|  | C10A1 |  |  |  |
|  | C12A1 |  |  |  |
|  | C13A1 |  |  |  |
|  | C14A1 |  |  |  |
|  | C15A1 |  |  |  |
|  | C16 A1 |  |  |  |
|  | C17A1 |  |  |  |
|  | C18A1 |  |  |  |
|  | C19A1 |  |  |  |
|  | C22A1 |  |  |  |
|  | C24A1 |  |  |  |
|  | C27A1 |  |  |  |
|  | C28A1 |  |  |  |
| *Stromal ECM* | ADIPOQ |  |  |  |
|  | AGRN |  |  |  |
|  | ASPN |  |  |  |
|  | BGN |  |  |  |
|  | BMPG |  |  |  |
|  | CTSD |  |  |  |
|  | DCN |  |  |  |
|  | DPT |  |  |  |
|  | EMILIN1 |  |  |  |
|  | FBN1 |  |  |  |
|  | FGA |  |  |  |
|  | FGB |  |  |  |
|  | FGG |  |  |  |
|  | FBLN1 |  |  |  |
|  | FBLN2 |  |  |  |
|  | FN1 |  |  |  |
|  | FLNA |  |  |  |
|  | LGALS1 |  |  |  |
|  | LGALS2 |  |  |  |
|  | LGALS3BP |  |  |  |
|  | LUM |  |  |  |
|  | OGN |  |  |  |
|  | PLG |  |  |  |
|  | POSTN |  |  |  |
|  | PRELP |  |  |  |
|  | PRG4 |  |  |  |
|  | PXDN |  |  |  |
|  | TNC |  |  |  |
|  | TNXB |  |  |  |
|  | VCAN |  |  |  |
| *BM* | C4A1 |  |  |  |
|  | C4A2 |  |  |  |
|  | C4A4 |  |  |  |
|  | C4A6 |  |  |  |
|  | HSPG |  |  |  |
|  | HSPG2 |  |  |  |
|  | LAMA1 |  |  |  |
|  | LAMA2 |  |  |  |
|  | LAMA3 |  |  |  |
|  | LAMA4 |  |  |  |
|  | LAMA5 |  |  |  |
|  | LAMB1 |  |  |  |
|  | LAMB2 |  |  |  |
|  | LAMB3 |  |  |  |
|  | LAMC1 |  |  |  |
|  | NID1 |  |  |  |
|  | NID2 |  |  |  |
|  |  |  |  |  |
|  | **Expressed** |  | **Not expressed** |  |
