# Supplementary material for: Increased peri-ductal collagen micro-organization may contribute to raised mammographic density
Source: Breast Cancer Res. 2016 Jan 8;18:5. doi: 10.1186/s13058-015-0664-2 (PMC4706673; doi:10.1186/s13058-015-0664-2)
Supplement: Additional file 3: Table S2. — Peptide frequencies for individual proteins identified in the mass spectrometry analysis. This table includes accession number, gene name and raw expression values for low and high mass spectrometry (MD) samples. (DOC 208 kb) [file 13058_2015_664_MOESM3_ESM.doc]

Table S2

| **Accession** | **Official Gene Symbol** | **Peptides used** | **Description** | **Expression in low** | **Expression in high** | **Log2 Fold Change** |
| --- | --- | --- | --- | --- | --- | --- |
| C9JF17 | APOD | 10 | Apolipoprotein D (Fragment) | 0.00207 | 0.01772 | 3.096 |
| P12273 | PIP | 10 | Prolactin-inducible protein | 0.00104 | 0.00855 | 3.041 |
| P01833 | PIGR | 12 | Polymeric immunoglobulin receptor | 0.00016 | 0.00094 | 2.595 |
| P25311 | AZGP1 | 14 | Zinc-alpha-2-glycoprotein | 0.00193 | 0.00540 | 1.489 |
| A6NDR9 | COL16A1 | 3 | Collagen alpha-1(XVI) chain | 0.00001 | 0.00003 | 1.319 |
| B1ALD9 | POSTN | 18 | Periostin | 0.00090 | 0.00212 | 1.239 |
| P01591 | IGJ | 6 | Immunoglobulin J chain | 0.00077 | 0.00165 | 1.097 |
| O43707 | ACTN4 | 5 | Alpha-actinin-4 | 0.00005 | 0.00011 | 1.062 |
| P01859 | IGHG2 | 4 | Ig gamma-2 chain C region | 0.00274 | 0.00483 | 0.821 |
| P02743 | APCS | 4 | Serum amyloid P-component | 0.00024 | 0.00039 | 0.719 |
| P14174 | MIF | 3 | Macrophage migration inhibitory factor | 0.00011 | 0.00018 | 0.693 |
| E9PNW4 | CD59 | 3 | CD59 glycoprotein | 0.00005 | 0.00008 | 0.568 |
| G8JLA8 | TGFBI | 3 | Transforming growth factor-beta-induced protein ig-h3 | 0.00042 | 0.00060 | 0.529 |
| P14543 | NID1 | 5 | Nidogen-1 | 0.00006 | 0.00009 | 0.525 |
| Q05707 | COL14A1 | 50 | Collagen alpha-1(XIV) chain | 0.01053 | 0.01493 | 0.504 |
| D6RGG3 | COL12A1 | 9 | Collagen alpha-1(XII) chain | 0.00620 | 0.00861 | 0.474 |
| E7EQB2 | LTF | 32 | Kaliocin-1 (Fragment) | 0.00512 | 0.00674 | 0.395 |
| B4E1Z4 | CFB | 8 | Complement factor B | 0.00107 | 0.00141 | 0.391 |
| P01009 | SERPINA1 | 26 | Alpha-1-antitrypsin | 0.01251 | 0.01631 | 0.383 |
| H7BYH4 | SOD1 | 4 | Superoxide dismutase [Cu-Zn] | 0.00011 | 0.00014 | 0.368 |
| P08572 | COL4A2 | 9 | Collagen alpha-2(IV) chain | 0.00071 | 0.00089 | 0.324 |
| Q9BXN1 | ASPN | 13 | Asporin | 0.00345 | 0.00426 | 0.302 |
| P05546 | SERPIND1 | 5 | Heparin cofactor 2 | 0.00158 | 0.00194 | 0.296 |
| O75083 | WDR1 | 5 | WD repeat-containing protein 1 | 0.00202 | 0.00248 | 0.292 |
| P02763 | ORM1 | 9 | Alpha-1-acid glycoprotein 1 | 0.00669 | 0.00815 | 0.284 |
| C9JC84 | FGG | 17 | Fibrinogen gamma chain | 0.00654 | 0.00788 | 0.269 |
| P02675 | FGB | 15 | Fibrinogen beta chain | 0.00588 | 0.00706 | 0.262 |
| P02748 | C9 | 4 | Complement component C9 | 0.00011 | 0.00013 | 0.252 |
| P01834 | IGKC | 6 | Ig kappa chain C region | 0.03153 | 0.03727 | 0.241 |
| B7TY16 | ACTN1 | 9 | Actinin alpha 1 isoform 3 | 0.00069 | 0.00081 | 0.224 |
| P01008 | SERPINC1 | 19 | Antithrombin-III | 0.00391 | 0.00453 | 0.213 |
| K7ER74 | APOC2 | 4 | Apolipoprotein C-II | 0.00045 | 0.00052 | 0.206 |
| Q8WZ42 | TTN | 29 | Titin | 0.01275 | 0.01462 | 0.197 |
| P20774 | OGN | 14 | Mimecan | 0.01685 | 0.01931 | 0.197 |
| P02461 | COL3A1 | 5 | Collagen alpha-1(III) chain | 0.01166 | 0.01321 | 0.181 |
| P51884 | LUM | 16 | Lumican | 0.04316 | 0.04823 | 0.160 |
| P02671 | FGA | 15 | Fibrinogen alpha chain | 0.00339 | 0.00376 | 0.151 |
| P02452 | COL1A1 | 18 | Collagen alpha-1(I) chain | 0.04660 | 0.05172 | 0.151 |
| P22352 | GPX3 | 3 | Glutathione peroxidase 3 | 0.00004 | 0.00005 | 0.148 |
| P04217 | A1BG | 15 | Alpha-1B-glycoprotein | 0.00136 | 0.00148 | 0.126 |
| E9PGN7 | SERPING1 | 8 | Plasma protease C1 inhibitor | 0.00039 | 0.00042 | 0.123 |
| P08603 | CFH | 29 | Complement factor H | 0.00127 | 0.00136 | 0.098 |
| P07585 | DCN | 17 | Decorin | 0.02424 | 0.02594 | 0.098 |
| G3V5I3 | SERPINA3 | 11 | Alpha-1-antichymotrypsin | 0.00192 | 0.00204 | 0.090 |
| P13671 | C6 | 5 | Complement component C6 | 0.00108 | 0.00113 | 0.066 |
| P09871 | C1S | 4 | Complement C1s subcomponent | 0.00010 | 0.00010 | 0.055 |
| P02768 | ALB | 20 | Serum albumin | 0.43351 | 0.43951 | 0.020 |
| P08185 | SERPINA6 | 3 | Corticosteroid-binding globulin | 0.00006 | 0.00006 | 0.019 |
| P35555 | FBN1 | 33 | Fibrillin-1 | 0.00142 | 0.00143 | 0.010 |
| P04075 | ALDOA | 7 | Fructose-bisphosphate aldolase A | 0.00097 | 0.00096 | -0.015 |
| Q3SY37 | CMA1 | 4 | CMA1 protein | 0.00029 | 0.00029 | -0.017 |
| P43652 | AFM | 10 | Afamin | 0.00058 | 0.00057 | -0.022 |
| P02787 | TF | 45 | Serotransferrin | 0.03470 | 0.03398 | -0.030 |
| C9JV77 | AHSG | 8 | Alpha-2-HS-glycoprotein | 0.00277 | 0.00266 | -0.056 |
| Q8IZC6 | COL27A1 | 5 | Collagen alpha-1(XXVII) chain | 0.00209 | 0.00199 | -0.068 |
| Q5W0H4 | TPT1 | 3 | Translationally-controlled tumor protein | 0.00004 | 0.00004 | -0.096 |
| P04003 | C4BPA | 8 | C4b-binding protein alpha chain | 0.00030 | 0.00028 | -0.097 |
| P23142 | FBLN1 | 3 | Isoform C of Fibulin-1 | 0.00017 | 0.00016 | -0.108 |
| P01024 | C3 | 58 | Complement C3 | 0.00946 | 0.00876 | -0.111 |
| P01023 | A2M | 41 | Alpha-2-macroglobulin | 0.00602 | 0.00553 | -0.122 |
| Q6UWY5 | OLFML1 | 4 | Olfactomedin-like protein 1 | 0.00019 | 0.00017 | -0.162 |
| P07339 | CTSD | 5 | Cathepsin D | 0.00252 | 0.00225 | -0.163 |
| P09382 | LGALS1 | 7 | Galectin-1 | 0.00160 | 0.00142 | -0.165 |
| H0Y7A7 | CALM2 | 4 | Calmodulin (Fragment) | 0.00018 | 0.00016 | -0.174 |
| P98160 | HSPG2 | 15 | Basement membrane-specific HS PGcore protein | 0.00027 | 0.00024 | -0.182 |
| E9PIT3 | F2 | 13 | Thrombin light chain | 0.00080 | 0.00070 | -0.200 |
| P08697 | SERPINF2 | 4 | Alpha-2-antiplasmin | 0.00015 | 0.00013 | -0.224 |
| P02790 | HPX | 19 | Hemopexin | 0.01377 | 0.01173 | -0.231 |
| P07237 | P4HB | 3 | Protein disulfide-isomerase | 0.00026 | 0.00022 | -0.237 |
| Q8IUX7 | AEBP1 | 3 | Adipocyte enhancer-binding protein 1 | 0.00005 | 0.00004 | -0.244 |
| P12110 | COL6A2 | 28 | Collagen alpha-2(VI) chain | 0.01048 | 0.00884 | -0.245 |
| P27797 | CALR | 3 | Calreticulin | 0.00068 | 0.00057 | -0.261 |
| P22105 | TNXB | 14 | Tenascin-X | 0.00027 | 0.00023 | -0.264 |
| P10909 | CLU | 8 | Clusterin | 0.00028 | 0.00023 | -0.279 |
| P21333 | FLNA | 46 | Filamin-A | 0.00331 | 0.00269 | -0.295 |
| P01042 | KNG1 | 16 | Isoform LMW of Kininogen-1 | 0.00305 | 0.00249 | -0.296 |
| P02749 | APOH | 10 | Beta-2-glycoprotein 1 | 0.00143 | 0.00116 | -0.304 |
| P04004 | VTN | 8 | Vitronectin | 0.00082 | 0.00066 | -0.313 |
| B1AVU8 | PSAP | 5 | Saposin-D | 0.00024 | 0.00019 | -0.339 |
| P02766 | TTR | 6 | Transthyretin | 0.01026 | 0.00797 | -0.363 |
| Q9Y490 | TLN1 | 9 | Talin-1 | 0.00016 | 0.00012 | -0.373 |
| P00918 | CA2 | 4 | Carbonic anhydrase 2 | 0.00009 | 0.00007 | -0.384 |
| E9PE77 | FN1 | 10 | Ugl-Y3 | 0.00051 | 0.00039 | -0.392 |
| F5H1A8 | GSN | 15 | Gelsolin | 0.00249 | 0.00190 | -0.394 |
| P07355 | ANXA2 | 19 | Annexin A2 | 0.00454 | 0.00345 | -0.395 |
| P36955 | SERPINF1 | 8 | Pigment epithelium-derived factor | 0.00032 | 0.00024 | -0.410 |
| E7ENL6 | COL6A3 | 110 | Collagen alpha-3(VI) chain | 0.04319 | 0.03232 | -0.418 |
| P02462 | COL4A1 | 4 | Collagen alpha-1(IV) chain | 0.00018 | 0.00013 | -0.420 |
| P11047 | LAMC1 | 8 | Laminin subunit gamma-1 | 0.00016 | 0.00012 | -0.458 |
| P08123 | COL1A2 | 8 | Collagen alpha-2(I) chain | 0.00935 | 0.00680 | -0.459 |
| P62937 | PPIA | 7 | Peptidyl-prolyl cis-trans isomerase A | 0.00219 | 0.00158 | -0.473 |
| Q5VY30 | RBP4 | 5 | Plasma retinol-binding protein(1-182) | 0.00042 | 0.00030 | -0.496 |
| A6NLG9 | BGN | 18 | Biglycan | 0.01173 | 0.00829 | -0.501 |
| P02760 | AMBP | 12 | Protein AMBP | 0.00052 | 0.00037 | -0.508 |
| P19652 | ORM2 | 7 | Alpha-1-acid glycoprotein 2 | 0.00385 | 0.00265 | -0.537 |
| P00747 | PLG | 11 | Plasminogen | 0.00072 | 0.00049 | -0.559 |
| A6NMZ7 | COL6A6 | 7 | Collagen alpha-6(VI) chain | 0.00126 | 0.00085 | -0.569 |
| P05997 | COL5A2 | 5 | Collagen alpha-2(V) chain | 0.00039 | 0.00026 | -0.589 |
| P13611 | VCAN | 7 | Versican core protein | 0.00036 | 0.00024 | -0.596 |
| P02750 | LRG1 | 5 | Leucine-rich alpha-2-glycoprotein | 0.00026 | 0.00017 | -0.621 |
| P0C0L4 | C4A | 25 | Complement C4-A | 0.00095 | 0.00061 | -0.630 |
| P01019 | AGT | 5 | Angiotensinogen | 0.00030 | 0.00019 | -0.634 |
| Q5T985 | ITIH2 | 10 | Inter-alpha-trypsin inhibitor heavy chain H2 | 0.00050 | 0.00032 | -0.637 |
| B7ZKJ8 | ITIH4 | 8 | 35 kDa inter-alpha-trypsin inhibitor heavy chain H4 | 0.00011 | 0.00007 | -0.647 |
| P04114 | APOB | 24 | Apolipoprotein B-100 | 0.01638 | 0.01046 | -0.647 |
| P51888 | PRELP | 15 | Prolargin | 0.00210 | 0.00131 | -0.683 |
| P08519 | LPA | 3 | Apolipoprotein(a) | 0.00967 | 0.00602 | -0.684 |
| P02652 | APOA2 | 4 | Apolipoprotein A-II | 0.00362 | 0.00224 | -0.688 |
| P08294 | SOD3 | 8 | Extracellular superoxide dismutase [Cu-Zn] | 0.00052 | 0.00032 | -0.735 |
| P12109 | COL6A1 | 31 | Collagen alpha-1(VI) chain | 0.02513 | 0.01475 | -0.769 |
| Q9Y6C2 | EMILIN1 | 4 | EMILIN-1 | 0.00008 | 0.00005 | -0.793 |
| P02747 | C1QC | 3 | Complement C1q subcomponent subunit C | 0.00235 | 0.00135 | -0.794 |
| Q6FHJ7 | SFRP4 | 3 | Secreted frizzled-related protein 4 | 0.00007 | 0.00004 | -0.848 |
| Q07507 | DPT | 7 | Dermatopontin | 0.00450 | 0.00244 | -0.885 |
| P18206 | VCL | 13 | Vinculin | 0.00034 | 0.00018 | -0.895 |
| E9PHK0 | CLEC3B | 6 | Tetranectin | 0.00023 | 0.00012 | -0.943 |
| P10809 | HSPD1 | 4 | 60 kDa heat shock protein, mitochondrial | 0.00009 | 0.00004 | -1.198 |
| P06727 | APOA4 | 10 | Apolipoprotein A-IV | 0.00189 | 0.00081 | -1.224 |
| P55268 | LAMB2 | 4 | Laminin subunit beta-2 | 0.00006 | 0.00003 | -1.232 |
| P06744 | GPI | 4 | Glucose-6-phosphate isomerase | 0.00005 | 0.00002 | -1.299 |
| P27169 | PON1 | 3 | Serum paraoxonase/arylesterase 1 | 0.00012 | 0.00005 | -1.310 |
| P00488 | F13A1 | 7 | Coagulation factor XIII A chain | 0.00074 | 0.00030 | -1.310 |
| P02647 | APOA1 | 25 | Apolipoprotein A-I | 0.02775 | 0.00860 | -1.691 |
| P04196 | HRG | 6 | Histidine-rich glycoprotein | 0.00116 | 0.00034 | -1.766 |
| F5H7E1 | ITIH1 | 5 | Inter-alpha-trypsin inhibitor heavy chain H1 | 0.00008 | 0.00002 | -1.797 |
| K7ERI9 | APOC1 | 3 | Truncated apolipoprotein C-I (Fragment) | 0.00007 | 0.00002 | -1.954 |
| P01031 | C5 | 5 | Complement C5 | 0.00003 | 0.00001 | -2.362 |
| P05164 | MPO | 13 | Myeloperoxidase | 0.00095 | 0.00009 | -3.408 |
